# Exploring determinants of COVID-19 vaccine uptake in Tanzania: A socio-ecological perspective

**DOI:** 10.4102/jphia.v16i3.709

**Published:** 2025-04-18

**Authors:** Ambrose T. Kessy, Chima E. Onuekwe, William M. Mwengee, Grace E. Saguti, Tumaini Haonga

**Affiliations:** 1 Directorate of Research, Publications and Consultancy, The University of Dodoma, Dodoma, United Republic of Tanzania; 2 Centre for Health and Allied Legal and Demographical Development, Research and Training (CHALADDRAT), Nnamdi Azikiwe University, Awka, Nigeria; 3 Department of Immunisation, Emergency Preparedness and Response, World Health Organization, Dar es Salaam, United Republic of Tanzania; 4 Department of Emergency Preparedness and Response, World Health Organization, Dar es Salaam, United Republic of Tanzania; 5 Health Promotion Unit, Ministry of Health, Dodoma, United Republic of Tanzania

**Keywords:** COVID-19, vaccine uptake, socio-ecological determinants, Tanzania, mixed-method study

## Abstract

**Background:**

The COVID-19 pandemic continues to challenge global public health, with vaccination playing a key role in mitigating transmission. Despite recognising its importance, Tanzania exhibits considerable regional disparities in vaccine uptake. Understanding the determinants influencing vaccination is essential.

**Aim:**

This study investigated determinants of COVID-19 vaccination rates within Tanzania, employing a socio-ecological framework to comprehensively examine individual, interpersonal, community, and institutional influences.

**Setting:**

Eight regions across Tanzania, purposively selected to represent urban, peri-urban, and rural contexts, reflecting varying socio-cultural and infrastructural conditions.

**Methods:**

A concurrent mixed-method design was utilised, combining quantitative surveys from 3098 participants with qualitative data collected through focus group discussions and key informant interviews.

**Results:**

Although general awareness of COVID-19 was notably high (99.3%), overall vaccine acceptance remained low (37.2%), exhibiting significant regional variations (22.5% in Morogoro to 50.0% in Mtwara). Individual factors such as personal vaccine beliefs, perceived safety, and misinformation significantly impacted uptake. Interpersonal influences from family, friends, and community leaders critically shaped vaccination decisions. Despite high acknowledgment of community leaders’ roles (88.3%), governmental campaign awareness was minimal (22.4%). Institutional factors, notably healthcare system trust and vaccine accessibility, also significantly influenced uptake.

**Conclusion:**

Findings advocate for region-specific, multilevel interventions addressing misinformation, engaging trusted community influencers, improving transparency, and enhancing healthcare service delivery to mitigate disparities and enhance vaccine acceptance.

**Contribution:**

The study offers insights foundational for tailored public health strategies, improving vaccine accessibility and resilience against future pandemics in Tanzania and comparable socio-ecological contexts.

## Introduction

COVID-19 pandemic has prompted global strategies to ensure vaccine uptake, with developed countries achieving high vaccination rates, while low- and middle-income countries (LMICs) such as Tanzania are lagging. This study delves into the socio-ecological factors influencing vaccine acceptance in seven diverse Tanzanian regions. The COVID-19 pandemic resulted in unprecedented morbidity and mortality worldwide, disrupting social, economic and healthcare systems.^1^ The development and subsequent global distribution of COVID-19 vaccines offer a robust means to combat the pandemic, curbing transmission and reducing disease severity.^2^ However, the success of these vaccines in controlling the pandemic depends largely on their acceptance and uptake by the global population.^3^

As of 12 November 2024, Tanzania has reported a cumulative total of 43 263 confirmed COVID-19 cases and 846 deaths.^4^ By April 2023, the country had administered approximately 39 392 419 vaccine doses, achieving a vaccination coverage of 51.0% of the total population.^5^ This marked a significant improvement from the 2.8% coverage reported in mid-January 2022. The increase in vaccination rates can be attributed to concerted efforts by the government and international partners to enhance vaccine delivery and address vaccine hesitancy.^5^ Despite these advancements, Tanzania’s vaccination coverage remains below the global target of 70% set by the World Health Organization (WHO). Challenges such as logistical constraints, misinformation and limited healthcare infrastructure continue to impede further progress. Ongoing initiatives aim to overcome these obstacles and increase vaccine uptake across the country. Despite access to vaccines through the COVID-19 Vaccines Global Access (COVAX) initiative, challenges, including vaccine hesitancy, logistics and distribution hurdles have impeded successful vaccination campaigns.^6^

Socio-ecological models, which consider the interplay between individual, interpersonal, community and societal factors, provide a useful lens to understand and address barriers to vaccination.^7^ Existing research underscores the importance of such an approach in examining health behaviours, including vaccination.^8^ In this study, we apply a socio-ecological framework to explore the determinants of COVID-19 vaccine uptake across eight regions in Tanzania: Arusha, Singida, Shinyanga, Tabora, Morogoro, Njombe, Mbeya and Mtwara. By elucidating these determinants, we aim to provide recommendations to enhance vaccine acceptance and uptake, contributing to the control of the pandemic in the Tanzanian context.

### The socio-ecological perspective

The socio-ecological perspective is a theoretical framework in environmental science, sociology, public administration and political science that provides a holistic understanding of the complex relationship between human societies and their natural environments, rooted in ecological thinkers such as Aldo Leopold and Garrett Hardin. However, it was the work of Urie Bronfenbrenner (1979) that laid the foundation for the modern socio-ecological perspective. Bronfenbrenner’s ecological systems theory highlighted the influence of various ecological levels, from microsystems (individuals and families) to macrosystems (society and culture), on human development.

Understanding the determinants of vaccine uptake is central to devising effective strategies for immunisation campaigns.^8^ The COVID-19 pandemic is a stark example of this. Despite the development and deployment of multiple effective vaccines,^9,10^ global vaccination rates remain unequal, with particularly low uptake in many LMICs.^6^ The socio-ecological model provides a comprehensive approach for understanding health behaviours, including vaccine uptake. This model posits that health behaviours are influenced by a complex interplay of factors at multiple levels – individual, interpersonal, community and societal.^7^ Several studies have successfully applied this model to understand the determinants of vaccine uptake, revealing how each of these levels contributes to the overall vaccine acceptance and coverage.^11^

Individual-level factors include demographic attributes, personal beliefs, knowledge and attitudes towards vaccination. For instance, research has identified a positive correlation between educational attainment and vaccine acceptance, with higher educated individuals more likely to accept vaccines.^12^ However, in Tanzania, the relationship between education and vaccine uptake may be nuanced because of issues of vaccine accessibility and misinformation. Interpersonal-level factors encompass social influences, such as family, friends and healthcare providers, which can greatly affect vaccine uptake. Healthcare providers, for instance, have a significant role in shaping patients’ attitudes towards vaccines.^13^ In Tanzania, social networks play an important role in health behaviours, particularly in rural communities. Traditional beliefs and community leaders significantly influence health behaviours. Community-level factors include community norms, trust in healthcare institutions and engagement, with trust being especially critical in Tanzania.^14^

Societal-level factors include larger structural determinants such as health policy, infrastructure and societal norms. The under-resourced healthcare system in Tanzania can create access barriers, influencing vaccine uptake. Inequitable vaccine distribution can also affect vaccine uptake, as seen in the current COVID-19 pandemic.^6^ Understanding the determinants of COVID-19 vaccine uptake in Tanzania requires a comprehensive approach that incorporates factors at multiple levels.^15^ By employing a socio-ecological perspective, this can help to gain a nuanced understanding of these factors and devise tailored strategies to enhance vaccine acceptance and coverage.

### An overview of COVID-19 uptake

Robinson et al.^16^ explored the factors contributing to vaccine hesitancy and reduced vaccine confidence in rural underserved populations, with a focus on COVID-19 vaccination. The study, conducted through qualitative methods, identified three main factors causing vaccine hesitancy: confidence, complacency and convenience. It emphasised the need to address these to boost vaccine uptake in rural underserved populations, emphasising the need for practical solutions and interventions to enhance public trust in immunisation systems. A large-scale global exploratory study done by de Figueiredo and Larson^17^ on the intent to accept COVID-19 vaccinations across 32 countries examined the levels of vaccine acceptance and explored socio-demographic determinants of acceptance. The study reveals variations in vaccine acceptance rates across countries, highlighting age, gender, education level and government perception of pandemic handling as factors influencing vaccine acceptance, emphasising the need for informed immunisation programmes.

A study in Anambra State, Nigeria,^18^ found that vaccine hesitancy is primarily because of misinformation, beliefs, conspiracy theories, a lack of knowledge and attitude. A total of 44.7% of respondents took the vaccine for protection, while 55.3% cited fear, not needing it and belief in no COVID-19 in Nigeria. The study suggests awareness creation, health education and government dispelling of rumours could help resolve vaccine hesitancy. A similar study was conducted by El-Ghitany et al.^19^ who collected data from 2919 participants through direct interviews using a structured questionnaire. The study shows a 66.5% acceptance rate for COVID-19 vaccination in Egypt, with reasons for refusal being mistrust of efficacy and safety concerns. Male gender, rural residence and lower education increase vaccination acceptance. Guardians of children and elderly individuals show higher acceptance rates.

The uptake of COVID-19 vaccines in Tanzania has been the subject of extensive research, revealing a complex interplay of factors influencing the decision-making process among healthcare workers (HCWs) and the general population.

### Vaccine hesitancy in Tanzania

Vaccine hesitancy in Tanzania remains a significant public health challenge despite concerted efforts to increase COVID-19 vaccination coverage. As of April 2023, approximately 51.0% of the Tanzanian population had been vaccinated against COVID-19, a notable increase from the 2.8% coverage reported in mid-January 2022.^5^ However, this still falls short of the WHO’s target of 70% coverage, indicating persistent hesitancy among the population. A recent study found that just over half of HCWs had received the COVID-19 vaccine, with a significant portion expressing hesitancy or outright refusal.^20^ This hesitancy was attributed to misinformation and inadequate knowledge about vaccine safety and efficacy. Similarly, a community-based survey indicated that confidence in COVID-19 vaccines was relatively low, with only 54.6% expressing confidence, which was significantly associated with vaccine uptake.^21^ Contradictions emerge when comparing these findings with global trends, where the acceptance and uptake rates of COVID-19 vaccination are reported to be 67.8% and 42.3%, respectively.^22^ The lower uptake in Tanzania, particularly among HCWs, is concerning given their role in public health and their potential to influence the general population’s perceptions. Moreover, the acceptance of COVID-19 vaccination for children in Tanzania was found to be low, with safety concerns being a major barrier.^23^ Interestingly, the uptake among people with type 2 diabetes in Tanzania was also suboptimal, influenced by factors such as education level, health insurance status and initial political hesitancy.^24^ This suggests that socio-economic factors and political climate play a significant role in vaccine uptake.

## Research methods and design

The study used a mixed-method approach to analyse socio-ecological factors affecting COVID-19 vaccine uptake, combining qualitative and quantitative data collection and analysis methods. Mixed-methods research is a comprehensive approach that integrates both quantitative and qualitative data collection methods within a single study, offering a more holistic understanding of complex phenomena.^25,26^ The design of mixed-method research typically falls into one of three categories: sequential, concurrent or embedded, each with distinct methodological characteristics and applications.^27,28^ This study employed a concurrent mixed-methods design, collecting quantitative and qualitative data simultaneously. This approach was chosen because it allowed for the comprehensive examination of socio-ecological factors affecting vaccine uptake within the same study period, capturing both the breadth of trends and the depth of underlying perceptions. This approach provided a good understanding of environmental, social and individual determinants, aiming to inform effective public health interventions and policies.

### Quantitative methods

Quantitative data were collected through structured surveys administered to a representative sample of participants across urban, peri-urban and rural areas. The survey included closed-ended questions designed to quantify factors such as awareness levels, trust in healthcare systems, perceived vaccine efficacy and safety and socio-demographic variables, including age, gender, education level and income. Statistical methods were employed to analyse these data, revealing correlations and patterns in vaccine uptake across different population subgroups.

#### Selection of study areas and participants

Three councils were selected from eight regions based on urbanity and rurality, with population size, geographical spread and accessibility as criteria. In total, 3098 participants were chosen through convenience sampling, considering time and resource constraints from the eight regions.

#### Sampling

The study was conducted between April 2023 and August 2023 across eight regions in Tanzania. These regions included Arusha, Singida, Shinyanga, Tabora, Morogoro, Njombe, Mbeya and Mtwara, which were purposively chosen to provide a comprehensive representation of the various experiences and perspectives across the seven administrative zones of Tanzania. The sampling process involved the selection of eight regions within Mainland Tanzania. The aim was to ensure that the findings of this study would be relevant and applicable to a wide range of areas within the country. Accordingly, three districts were selected to participate in each region giving a total of 24 districts (Table 1). Districts were purposively selected to represent an urban, semi-urban and a rural district. In each district, two wards (one urban, one rural) were purposively selected where a minimum of 60 households were randomly selected from each ward to participate in the study.

**TABLE 1:** Geographical distribution of the mini-survey.

S/No	Region	District/council	Total (*n*)
1	Arusha	Arusha CC	107
		Arusha DC	90
		Meru DC	161
		Total	358
2	Mbeya	Chunya DC	141
		Kyela DC	150
		Mbeya CC	140
		Total	431
3	Morogoro	Ifakara DC	77
		Morogoro DC	141
		Morogoro MC	147
		Total	365
4	Mtwara	Mtwara DC	135
		Mtwara MC	142
		Nanyamba DC	143
		Total	420
5	Njombe	Makambako TC	103
		Makete DC	121
		Njombe TC	167
		Total	391
6	Shinyanga	Kishapu DC	124
		Shinyanga DC	124
		Shinyanga MC	121
		Total	369
7	Singida	Ikungi DC	126
		Singida DC	132
		Singida MC	128
		Total	386
8	Tabora	Tabora MC	137
		Urambo DC	121
		Uyui DC	120
		Total	378

-	-	**Grand total**	**3098**

CC, City Council; DC, District Council; MC, Municipal Council; S/No, serial number; TC, Town Council.

#### Data collection methods

The primary tool for quantitative data collection was a structured survey questionnaire. This instrument was developed based on a thorough review of existing literature on vaccine hesitancy and socio-ecological determinants, as well as input from local healthcare professionals and public health experts. The survey consisted of closed-ended questions, grouped into thematic sections to assess: awareness and knowledge, attitudes and perceptions, behavioural and structural factors and demographic and socio-economic variables.

Data collection was conducted by trained field staff fluent in local languages, ensuring that respondents could fully comprehend and respond to the questions. Surveys were administered both in person and digitally where feasible, allowing for greater reach and inclusivity. To ensure data quality, the field team underwent rigorous training in ethical data collection practices, survey administration and respondent engagement. Moreover, pilot testing of the survey instrument was conducted in a non-study region to identify and rectify any ambiguities or biases in question design. The collected data were systematically entered into a secure database and cross-checked for accuracy and completeness. Statistical software, Statistical Package for Social Sciences (SPSS) (IBM SPSS Statistics, Armonk, New York, United States [US]), was employed for data cleaning and analysis.

#### Quantitative data analysis

The quantitative data analysis focussed on providing a descriptive statistical overview of the factors influencing COVID-19 vaccine uptake in Tanzania. The primary objective was to summarise and visualise the collected data to identify patterns and trends across different demographic and socio-ecological groups. Statistical analysis was conducted using SPSS ensuring systematic and reliable data handling. The summarised data were systematically presented in tables, allowing for easy interpretation and comparison of findings. Tables also highlighted key differences between demographic groups, such as the variation in regions.

### Qualitative methods

Qualitative data were gathered through key informant interviews (KIIs) and focus group discussions (FGDs) with key stakeholders, including community leaders, HCWs and individuals who had either received or declined vaccination. These interviews explored deeper socio-cultural and psychological factors, such as perceptions of vaccine safety, religious and traditional beliefs, the influence of misinformation and experiences with healthcare services. The qualitative approach allowed for the exploration of lived experiences and the identification of barriers and facilitators to vaccine uptake that quantitative surveys may not capture. Data were analysed using thematic analysis, where transcripts were coded to identify recurring themes and narratives. This process provided rich contextual insights into how individual and collective perceptions shape vaccine behaviour.

#### Selection of study areas

Within each region, specific districts and communities were selected to provide a balanced representation of urban, peri-urban and rural settings. This stratification ensured that the study captured the unique challenges and opportunities associated with vaccine uptake in each type of settlement. The selection process also considered logistical feasibility, including the accessibility of areas for the research team, as well as the availability of local collaborators to facilitate data collection. This approach ensured that the study areas provided a comprehensive and contextually relevant understanding of vaccine uptake across Tanzania.

#### Sampling

The sampling process for the qualitative study was designed to ensure the inclusion of diverse voices and perspectives from the selected study areas. A purposive sampling strategy was employed, guided by the need to capture a wide range of experiences and socio-cultural contexts related to COVID-19 vaccine uptake. Participants were recruited from the eight selected regions, with efforts made to include individuals from various socio-demographic backgrounds. Accordingly, 42 KII and 48 FGD participants drawn from two wards in three districts from each region formed the sample size for the study per region. The inclusion criteria were designed to ensure representation from key stakeholder groups, including HCWs, community leaders: vaccinated and unvaccinated individuals, marginalised groups.

#### Data collection

Qualitative data collection consisted of two primary methods: FGDs and KIIs. Six FGDs were conducted in each region, with 8–12 participants per group. A total of 24 FGDs from eight regions were utilised to gather insights into community perspectives, social norms and collective experiences related to COVID-19 vaccination. In parallel, six KIIs were conducted in each region making a total of 144 key informants. Key informants included healthcare professionals, community leaders and local policymakers. Key informant interviews were instrumental in gaining insights into the local health infrastructure, policies and societal level factors influencing vaccine uptake.

#### Qualitative data analysis

The qualitative data collected through KIIs and FGDs underwent a systematic process of transcription, translation and thematic analysis. This method ensured a structured yet flexible framework for identifying, analysing and interpreting patterns of meaning within the data. All audio recordings of interviews and discussions were transcribed verbatim, preserving the richness and context of participants’ responses. The translations were conducted to render the data into English while maintaining the original intent and cultural nuances of the participants’ narratives. A bilingual team of researchers cross-verified the translations to ensure accuracy and fidelity to the original meanings. Using NVivo software (Lumivero, Burlington, Massachusetts, US), researchers assigned labels to significant segments of text that captured key ideas, sentiments or behaviours related to vaccine uptake. Both inductive and deductive coding techniques were employed – inductive to allow themes to emerge directly from the data and deductive to align findings with the study’s conceptual framework. Coded data were reviewed to identify overarching themes and subthemes, representing broader patterns and recurring narratives. The themes were reviewed for coherence and relevance, ensuring that they captured the complexity and depth of the participants’ perspective. Finally, themes were organised into a narrative that illustrated the socio-ecological dimensions of vaccine uptake. Supporting quotations were selected to exemplify key findings, adding authenticity and depth to the analysis.

### Ethical considerations

Ethical considerations were foundational to the study, ensuring participants’ rights, dignity and well-being were upheld throughout the research process. The ethical approval for the study was obtained from the University of Dodoma (UDOM)’s Institutional Research Review Committee of the University of Dodoma (UDOM) (MA.84/261/76/214). Participants were fully informed about the study’s purpose, procedures and their rights, with consent obtained in either written or verbal form, depending on literacy levels. Confidentiality was rigorously maintained through secure data storage, anonymisation of responses and limited access to identifiable information. Cultural sensitivity was prioritised by engaging community leaders and aligning research practices with local norms, ensuring that discussions on sensitive topics, such as vaccine hesitancy and healthcare trust, were conducted respectfully.

Efforts were made to mitigate potential risks, such as emotional distress, by fostering a supportive environment and providing referrals to healthcare services where needed. Special attention was given to marginalised groups, such as rural residents and women, to ensure diverse representation. Participants were treated with autonomy and respect, with the freedom to withdraw or refrain from answering certain questions. In reporting, the study emphasised accuracy and avoided stigmatisation or misrepresentation, presenting findings in a way that maintained the integrity of the research. This ethical framework not only safeguarded participants but also strengthened the credibility of the findings, making them relevant for informing culturally sensitive and equitable public health interventions.

## Results

Our research identified and examined four key levels of influence on vaccine uptake: (1) individual level, (2) interpersonal level, (3) community level and (4) institutional level.

### Intrapersonal perspectives on COVID-19

The study of intrapersonal perspectives on COVID-19 examines individuals’ awareness, beliefs, perceptions of symptoms and potential consequences. It aims to gain a comprehensive understanding of how individuals perceive and respond to the global health crisis. The findings show that 99.3% of the population is aware of the COVID-19 pandemic, indicating effective communication and public health initiatives. However, a small percentage, 0.7%, remains uninformed. Geographical differences exist, with Arusha and Tabora having complete awareness; Mbeya, Mtwara, Njombe, Shinyanga and Singida having nearly universal awareness and Morogoro having the lowest yet highest awareness rate at 97.5% (Table 2).

**TABLE 2:** Awareness of the COVID-19 pandemic (*N* = 3098).

Region	Have you heard about COVID-19 pandemic?		Total (%)
	No (%)	Yes (%)	
Arusha	-	100.0	100.0
Mbeya	0.7	99.3	100.0
Morogoro	2.5	97.5	100.0
Mtwara	0.5	99.5	100.0
Njombe	0.8	99.2	100.0
Shinyanga	0.8	99.2	100.0
Singida	0.3	99.7	100.0
Tabora	-	100.0	100.0

**Total**	**0.7**	**99.3**	**100.0**

Qualitative responses complement these findings, illustrating reliance on traditional media (television, radio) and social media for information. The response from a member in a KII at Mkambarani, Morogoro District Council, Morogoro Region, highlights the role of media in disseminating information about COVID-19 and emphasises the pandemic’s unique impact on both individual and national levels, including economic repercussions:

‘For the first time on the issue of Corona, I heard it on media including radio and television. But it felt like a repetition because they were saying that it had occurred in previous years, except this came a bit differently in that it affected a broader region, or a higher percentage of people were harmed. People were harmed, and even the economy of individuals was shaken, not just that of the nation, because those activities that gathered people were [*affected*].’ (KII9, Council Official, Female, 52 years, Morogoro District Council, Morogoro Region)

Table 3 shows significant geographical variations in vaccination rates, with Mtwara and Singida having the highest rates at 50.0% and 49.7%, respectively. Morogoro and Mbeya have the lowest rates at 22.5% and 26.2%, respectively. Other regions, including Arusha, Njombe, Shinyanga and Tabora, have rates between 36.6% and 37.7%, which are not as low as those in Morogoro and Mbeya.

**TABLE 3:** COVID-19 vaccination status (*N* = 3098).

Region	Have you been vaccinated against COVID-19?		Total (%)
	No (%)	Yes (%)	
Arusha	62.6	37.4	100.0
Mbeya	73.8	26.2	100.0
Morogoro	77.5	22.5	100.0
Mtwara	50.0	50.0	100.0
Njombe	63.4	36.6	100.0
Shinyanga	62.3	37.7	100.0
Singida	50.3	49.7	100.0
Tabora	63.2	36.8	100.0

**Total**	**62.8**	**37.2**	**100.0**

Reasons for not getting vaccinated include mixed messaging and concerns about the vaccine’s effects on specific groups, as highlighted in FGDs. For example, a response from a participant in an FGD at Kambaragwe Ward, Shinyanga Region, reveals a reliance on alternative preventive measures against COVID-19:

‘I haven’t received the vaccine because I’ve been adhering to other preventive measures against COVID-19, such as practicing good hygiene and avoiding crowded places. Due to these precautions, I haven’t felt the urgency to get vaccinated and have continued to prioritize these measures.’ (FGD2, Small Farmer, Male, 23 years, Shinyanga)

### Interpersonal influence and COVID-19

Interpersonal influence on COVID-19 vaccination is influenced by individual beliefs, values, attitudes, societal norms, cultural practices, safety concerns, healthcare resource access and accurate information, resulting in shared opinions and decisions. Trusted sources of COVID-19 information significantly influence public opinion and vaccination decisions, ensuring individuals have access to reliable information from credible sources such as government agencies, healthcare providers and scientific experts.

The findings from FGDs and KIIs show the role of social networks is involved in the decision-making process. People’s reliance on different media sources and community discussions likely had an impact on their perceptions and actions regarding COVID-19 and vaccination. The findings show that there are important connections between views on COVID-19 vaccination and trust in information more broadly. For example, a response from a participant in FGD at Karatu Ward, Karatu District Council, Arusha Region, reflects a proactive and community-oriented approach to COVID-19 vaccination, underlining both personal and public health motivations:

‘Yeah, I got vaccinated because I was motivated and I saw that the way I move around and meet many people I could get infected, so I had to get vaccinated. But also, I motivated my family, my wife to get vaccinated, but another reason is that if I got it [*the virus*], I wouldn’t want to infect others, so I saw it necessary to get vaccinated.’ (FGD5, Village Executive Officer, Female, 35 years, Karatu, Arusha)

### Community response to COVID-19

The community’s response to COVID-19 has been diverse, with some scepticism and others embracing vaccination. Prevention measures include social distancing guidelines and hygiene protocols. Community leaders have raised awareness and promoted vaccination through outreach strategies. The findings show that community leaders effectively educate the public about COVID-19 immunisation, with 88.3% of participants expressing active participation. However, geographical disparities exist, with 24.4% of respondents in Morogoro not receiving communication from their leaders (Table 4).

**TABLE 4:** Information provided by community leaders on the importance of COVID-19 vaccination (*N* = 3098).

Region	Have your community leaders (political, religious, community) informed you about the importance of vaccination against COVID-19?		Total (%)
	No (%)	Yes (%)	
Arusha	9.5	90.5	100.0
Mbeya	10.0	90.0	100.0
Morogoro	24.4	75.6	100.0
Mtwara	8.1	91.9	100.0
Njombe	9.7	90.3	100.0
Shinyanga	10.6	89.4	100.0
Singida	10.6	89.4	100.0
Tabora	11.4	88.6	100.0

**Total**	**11.7**	**88.3**	**100.0**

The study highlights influential groups in public health communication, including religious bodies, politicians and community leaders, and their impact on public perception and actions towards health initiatives. The response from a KII with a leader of a local association in Shinyanga district council, Shinyanga region, highlights the significant influence of religious leaders, particularly from Christian communities, in disseminating public health information during the COVID-19 pandemic:

‘That is, leaders who have a great influence are these leaders coming from Christianity. They have had a great influence on the community, because even when this COVID vaccination began, they were given leaflets and announced in their places of worship to deliver the message to the Isela Magazi community along with mosques.’ (KII8, Leader Local Association, Male, 40 years, Shinyanga)

### Institutional policies and COVID-19

Institutions have implemented policies to prevent and respond to COVID-19, including prevention measures, response protocols and coping resources, continuously evaluating effectiveness and providing counselling and financial assistance. Table 5 shows modest awareness levels of governmental initiatives to promote COVID-19 vaccination, with 22.4% of participants in all areas showing a deficiency in information transmission. Geographical variations in awareness levels are evident, with Mbeya and Mtwara having the lowest levels at 6.0% and 6.2%, respectively. Arusha and Singida have the highest levels at 37.0% and 39.4%, respectively.

**TABLE 5:** Government measures supporting COVID-19 vaccination (e.g., vaccination policies for travelers) (*N* = 3098).

Region	Do you know of any government measure in support of COVID-19 vaccination? (e.g., policy on vaccination for travellers)		Total (%)
	No (%)	Yes (%)	
Arusha	60.6	39.4	100.0
Mbeya	94.0	6.0	100.0
Morogoro	85.5	14.5	100.0
Mtwara	93.8	6.2	100.0
Njombe	76.7	23.3	100.0
Shinyanga	71.5	28.5	100.0
Singida	63.0	37.0	100.0
Tabora	71.4	28.6	100.0

**Total**	**77.6**	**22.4**	**100.0**

## Discussion

The study on the socio-ecological factors influencing COVID-19 vaccine uptake in Tanzania reveals varied parental vaccine intentions, highlighting the need for a multilevel analysis encompassing intrapersonal perspectives, community responses, institutional policies and societal impacts to inform effective public health interventions.

### Navigating intrapersonal factors to enhance vaccine uptake

The study reveals that personal beliefs and perceived risks significantly influence vaccine hesitancy, necessitating the development of tailored communication strategies to boost vaccine acceptance. The findings highlight high awareness levels of COVID-19 among Tanzanians. However, despite this awareness, there are disparities in vaccine uptake, which are influenced by individual factors such as perceptions of vaccine safety and efficacy, gender, education and age. This suggests that while awareness is high, there are still gaps in knowledge or trust regarding the vaccine, which impacts personal decisions to get vaccinated. At the individual level, knowledge and perceptions about vaccine efficacy and safety were identified as critical determinants. While individual factors play a role in vaccine hesitancy, focussing solely on personal beliefs and perceptions may overlook systemic issues that contribute to low uptake rates. Structural barriers, such as limited access to healthcare facilities or inadequate vaccine distribution infrastructure, could be equally significant in determining vaccination rates.^29^ Moreover, the emphasis on tailored communication strategies might inadvertently downplay the importance of addressing broader societal concerns and misinformation that fuel vaccine hesitancy on a larger scale. These results mirror global findings highlighting the role of perceived vaccine safety and efficacy in shaping vaccination decisions.^30^

Misinformation, which was rampant in our study regions, also played a significant role. These misconceptions were fuelled by social media, aligning with worldwide observations about the damaging role of misinformation in vaccine uptake.^31^ The findings underscore the influence of interpersonal factors on vaccine uptake, particularly the role of social networks. Individuals embedded in pro-vaccine networks were more likely to be vaccinated, underscoring the power of social influence on health behaviours.^32^ Respected community figures, including religious leaders and local authorities, also played a crucial role. Similar observations were made in polio vaccination campaigns in Nigeria, where engagement with religious leaders improved vaccine acceptance.^18^ The findings highlight the critical importance of addressing misinformation and leveraging social networks to improve vaccine acceptance.^33^ By engaging respected community figures and harnessing the power of pro-vaccine social circles, public health initiatives can more effectively overcome vaccine hesitancy and increase immunisation rates.

### Strengthening community engagement for enhanced vaccine uptake in Tanzania

Community responses to the COVID-19 vaccine in Tanzania vary significantly. The variation in the adherence rates to vaccine advice, as well as the differences in reasons for non-compliance, points to a need for more localised and tailored public health communication strategies. Community-based efforts, such as those employed by the Community Vaccine Collaborative (CVC) in Pittsburgh, could be beneficial. The CVC’s approach of promoting vaccine equity, addressing mistrust and involving community health workers could serve as a model for Tanzania, particularly in reaching underrepresented and rural populations.^34^ Community engagement and trust are essential for vaccine uptake. Community-based approaches, such as the CVC’s initiative in Pittsburgh, demonstrate the effectiveness of involving community leaders and health workers in promoting vaccine equity and addressing mistrust.^34^

In Tanzania, the variation in vaccine adherence and reasons for non-compliance across communities underscores the need for localised health communication strategies.^35^ At the community level, trust in health institutions emerged as a vital determinant of vaccine uptake. This aligns with previous studies linking healthcare trust to vaccine acceptance.^36^ Regions such as Arusha and Morogoro, which have stronger health infrastructures, reported higher vaccination rates, emphasising the necessity of robust local health systems in supporting vaccine distribution and administration. These findings elucidate the pivotal role of community-driven approaches and local health system strengthening in enhancing vaccine uptake and equity. Through the cultivation of trust and the adaptation of communication strategies to specific communities, public health initiatives can more efficaciously address vaccine hesitancy and augment overall immunisation rates.^37,38^

### Optimising vaccine policies for diverse populations in Tanzania

The findings have shown that institutional policies play a critical role in vaccine uptake. In Tanzania, demographic factors such as gender, education, religion and age significantly correlate with vaccination status. This indicates the necessity for policies that are not only broad reaching but also tailored to address the specific needs and concerns of different demographic groups. In addition, the Tanzanian government’s efforts, such as the Initiative for Global Vaccine Access (Global VAX) and the Accelerated Community-Based COVID-19 Vaccination Strategy, aim to increase vaccine accessibility and uptake, particularly among women and younger people.^35^ These initiatives demonstrate the government’s recognition of the need for targeted approaches in vaccine distribution. However, critics argue that tailoring vaccine policies to specific demographic groups may lead to unintended consequences, such as reinforcing existing social divisions or creating perceptions of favouritism.^39,40^ Some experts contend that a more unified, universal approach to vaccine distribution could be more equitable and efficient, potentially avoiding the complexities and resource demands of implementing multiple targeted strategies.

In response to the pandemic, governments worldwide are actively working to vaccinate their populations against COVID-19.^40,41^ Different countries are using various approaches in vaccine distribution and administration. Some governments are providing vaccines for free or at subsidised prices to encourage uptake.^3^ These differing approaches to vaccine distribution have led to varying levels of success in immunisation campaigns across countries. Some countries have implemented innovative strategies, such as mobile vaccination units or door-to-door campaigns, to reach remote or underserved populations. Moreover, public-private partnerships have emerged in some regions to accelerate vaccine rollout and address logistical challenges in distribution and administration.^42^

### Institutional policies to enhance vaccine uptake across diverse demographics

On a societal level, vaccine hesitancy in Tanzania is shaped by socio-political factors, misinformation and deeply ingrained myths about vaccines. Concerns about vaccine safety, inconsistent messaging from community and political leaders and a preference for traditional remedies contribute to hesitancy. Health promotion messages must clearly respond to the diverse public concerns regarding vaccine misinformation and safety, while strategies aimed at improving vaccine uptake should also carefully navigate the complexities posed by pandemic politicisation as well as Tanzania’s distinct cultural and social contexts.^43^ Societal level factors, particularly vaccine availability and distribution, significantly influenced vaccine uptake. Remote regions such as Singida and Shinyanga experienced lower vaccination rates because of logistical challenges. Similar findings have been reported in other LMICs, where geographic accessibility is a critical barrier to vaccination.^44^

The findings underscore the importance of addressing socio-ecological factors influencing vaccine uptake in Tanzania, including strengthening health infrastructure, ensuring equitable distribution and fostering community trust in health institutions. A similar approach to exploring these multilevel influences has been used to understand vaccine uptake in other global contexts.^8^ Overall, societal impacts and responses to COVID-19 continue to evolve as we learn more about this disease and how best to manage it. Governments and individuals alike must work together towards finding effective solutions that will help us navigate this challenging time successfully.

### Limitations of the study

The study faced several limitations that could impact the generalisability and comprehensiveness of its findings. Firstly, the use of convenience sampling in the regions may have introduced selection bias, potentially overrepresenting more accessible populations while underrepresenting marginalised or hard-to-reach groups, such as nomadic communities. Secondly, logistical and resource constraints limited the scale of data collection, which may have excluded some remote areas with unique socio-ecological challenges affecting vaccine uptake. Moreover, the concurrent mixed-methods design, while efficient, may not have allowed for the iterative refinement of data collection tools that sequential designs could have facilitated. These limitations underscore the need for cautious interpretation of the findings and highlight areas for improvement in future.

## Conclusion

This study elucidates the multifaceted determinants of COVID-19 vaccine uptake in Tanzania, revealing significant regional disparities and influences across individual, interpersonal, community and institutional levels. Despite high awareness of COVID-19, vaccination rates remain low, primarily because of personal beliefs, misinformation and perceived risks affecting individual decisions. Interpersonal influences from family, friends and trusted community and religious leaders critically shape attitudes toward vaccination. Community engagement proves pivotal; however, gaps in awareness of governmental initiatives indicate a need for enhanced communication strategies.

Improving vaccination rates necessitates a holistic and multilevel approach. Tailored interventions should directly address individual concerns by providing accurate information to counteract misinformation and build confidence in vaccine safety and efficacy. Leveraging trusted local influencers can enhance interpersonal and community-level engagement, fostering a supportive environment for vaccine acceptance. Institutional efforts must focus on increasing transparency in vaccine distribution, improving accessibility and strengthening healthcare infrastructure, especially in remote regions. Collaborative endeavours among policymakers, public health officials, community leaders and international partners are vital to overcome logistical challenges and ensure equitable vaccine distribution. Strategies such as deploying mobile clinics and involving community health workers can effectively reach underserved populations.

Addressing regional disparities requires region-specific strategies that consider local socio-cultural contexts. Investment in healthcare infrastructure and capacity-building is crucial to support sustained vaccination efforts and enhance overall public health resilience. By implementing multilevel, tailored interventions and fostering cross-sector collaboration, it is possible to improve vaccination rates, reduce regional disparities and contribute significantly to controlling the pandemic in Tanzania.
